# An initial application of computerized adaptive testing (CAT) for measuring disability in patients with low back pain

**DOI:** 10.1186/1471-2474-9-166

**Published:** 2008-12-18

**Authors:** Atilla Halil Elhan, Derya Öztuna, Şehim Kutlay, Ayşe A Küçükdeveci, Alan Tennant

**Affiliations:** 1 Department of Biostatistics, Faculty of Medicine, Ankara University, Ankara, Turkey; 2 Department of Physical Medicine and Rehabilitation, Faculty of Medicine, Ankara University, Ankara, Turkey; 3 Department of Rehabilitation Medicine, Faculty of Medicine and Health, University of Leeds, Leeds, UK

## Abstract

**Background:**

Recent approaches to outcome measurement involving Computerized Adaptive Testing (CAT) offer an approach for measuring disability in low back pain (LBP) in a way that can reduce the burden upon patient and professional. The aim of this study was to explore the potential of CAT in LBP for measuring disability as defined in the International Classification of Functioning, Disability and Health (ICF) which includes impairments, activity limitation, and participation restriction.

**Methods:**

266 patients with low back pain answered questions from a range of widely used questionnaires. An exploratory factor analysis (EFA) was used to identify disability dimensions which were then subjected to Rasch analysis. Reliability was tested by internal consistency and person separation index (PSI). Discriminant validity of disability levels were evaluated by Spearman correlation coefficient (r), intraclass correlation coefficient [ICC(2,1)] and the Bland-Altman approach. A CAT was developed for each dimension, and the results checked against simulated and real applications from a further 133 patients.

**Results:**

Factor analytic techniques identified two dimensions named "body functions" and "activity-participation". After deletion of some items for failure to fit the Rasch model, the remaining items were mostly free of Differential Item Functioning (DIF) for age and gender. Reliability exceeded 0.90 for both dimensions. The disability levels generated using all items and those obtained from the real CAT application were highly correlated (i.e. > 0.97 for both dimensions). On average, 19 and 14 items were needed to estimate the precise disability levels using the initial CAT for the first and second dimension. However, a marginal increase in the standard error of the estimate across successive iterations substantially reduced the number of items required to make an estimate.

**Conclusion:**

Using a combination approach of EFA and Rasch analysis this study has shown that it is possible to calibrate items onto a single metric in a way that can be used to provide the basis of a CAT application. Thus there is an opportunity to obtain a wide variety of information to evaluate the biopsychosocial model in its more complex forms, without necessarily increasing the burden of information collection for patients.

## Background

Low back pain (LBP) is a frequently reported musculoskeletal problem causing much disability [1]. The economic burden of LBP on the society is great due to both its high prevalence and chronicity [2]. The main goals in the management of LBP are to control pain, maintain and improve function and consequently prevent disability [3]. Thus the assessment of disability is essential for both planning and monitoring therapeutic interventions. There are many questionnaires available to assess disability for outcome measurement in LBP [4,5] and most recently, 'core sets' of items have been proposed based upon the International Classification of Functioning, Disability and Health (ICF) [6].

The ICF, developed by the World Health Organization (WHO), aims to provide a unified and standard language and framework for the description of health and health-related conditions [7]. It describes a model which systematically classifies the health and health related domains into two components: 1) body functions and structures; 2) activities and participation. According to this model, functioning is an umbrella term encompassing all body functions, activities and participation; similarly disability is an umbrella term including both impairments and activity limitations or participation restriction. Impairments catalogue the problems in body structure (e.g. displacement of vertebral disks) or body functions (e.g. pain in back) such as a significant deviation or loss. Activity is defined as the execution of a task or action by an individual whereas participation is involvement in a life situation. Activity limitations are difficulties an individual may have in executing such activities. Participation restrictions are problems an individual may have experienced in involvement in life situations. The ICF also lists environmental factors that interact with functioning and disability as contextual factors. The unit of classification in ICF is called as 'category'. Within each component, there are various individual categories arranged in a stem/branch/leaf scheme. In order to capture the integration of various aspects of functioning, ICF uses a biopsychosocial approach including biological, individual and social perspectives [7]. Impairments such as displacement of vertebral disks or pain in back can cause limitations in individual activities such as dressing, or walking and/or restriction in societal participation such as work or leisure. These domains may be further mediated by environmental factors such as terrain, or the provision of assistive devices.

Clinicians and other health professionals could be faced with using a substantive range of outcome measures if even part of the ICF model is to be routinely implemented. This potentially presents a considerable burden to patients, as well as a formidable administrative burden to hard pressed health care professionals. One solution for this problem is to make use of a relatively new approach to outcome measurement, built upon existing work, such that patient and professional burden can be reduced or, where necessary, information collected can be increased at no extra burden. The mechanism by which this solution can be obtained is to implement a Computerized Adaptive Testing (CAT) approach for measuring disability in LBP. CAT, an outcome measurement approach for comprehensive and precise assessment of patient-related outcomes, is being used with increasing frequency in the health care field [8-15]. The approach uses a computer to administer test items to patients. In doing so, using a previously calibrated set of items called an item bank, it selects the most informative items for each individual patient according to their level on the construct being measured [16]. This avoids the administration of a large number of questionnaire items by selecting items close to the person's ability level, effectively constructing a "tailored test" for each individual. The CAT approach allows for the collection of precise outcome information that can simply be applied in both clinical and research settings [8,16-18].

Thus the CAT approach depends on a calibrated set of item difficulties, the calibrations of which are derived from a particular Item Response Theory (IRT) model [17,19-21]. This calibration and the associated item information derived are the most important elements in CAT applications [10,22]. IRT models are statistical models that describe the probability of choosing each response on a questionnaire item as a function of the construct (latent trait) being measured [16,23]. With IRT, item calibrations and person estimates are located on the same metric. As such, the items are inherently linked to the metric both in terms of ability of the person and the amount of information that an item provides at each point along the trait. This property supports an efficient selection of items during a CAT administration. Thus the combination of IRT and CAT creates considerable flexibility in administering tests in an adaptive approach for each patient [8].

Several recent studies have reported the use of CAT in lumbar spine disorders. In the earliest study, Hart et al. developed a CAT assessing lumbar functional status in terms of activities domain of the ICF [12]. Similarly in another study CAT was applied to measure the self-care and mobility activities in an orthopaedic outpatient physical therapy setting [8]. Most recently, Kopec et al. used a CAT program to measure 5 domains of health-related quality of life: daily activities, walking, handling objects, pain and feelings [9]. To our knowledge, no study has yet reported the use of a CAT program assessing disability in LBP in a comprehensive manner as defined in the ICF.

Therefore the aim of this study was to explore the potential of CAT for measuring disability in patients with LBP based on the definition of disability in ICF which includes impairments, activity limitation, and participation restriction. In order to achieve this aim, item banks were developed from currently used questionnaires. The internal construct validity of each item bank was examined by testing the assumptions of unidimensionality, local independence and Differential Item Functioning (DIF) by age and gender, within the framework of the Rasch measurement model [24]. CAT software was then developed to utilise the calibrated items from each item bank. Real and simulated CAT applications were applied and the correlation between the disability levels generated by CAT, and the responses to all items in the item bank, was determined. Finally convergent validity between the CAT derived estimates and the scores from each original questionnaire were examined.

## Methods

### Patients and setting

Data was collected in the Department of Physical Medicine and Rehabilitation at the Medical Faculty of Ankara University, Turkey, from February 2007 to November 2007. A total of 399 outpatients with low back pain were included in the study. Patients with non-mechanical back pain resulting from inflammatory, infectious, malignant or visceral diseases were excluded. In the first stage of the study 266 patients answered all the questions in the total item set obtained from the selected questionnaires (given below). After development of the item banks, the second stage involved another group of 133 patients completing the item banks (items determined after Rasch analysis) under a CAT version and by 'paper and pencil'.

In all cases, questionnaires were either self-completed by literate patients, or where patients were illiterate, the questionnaires were administered by one of the authors (DÖ). At the CAT stage, the same author also helped the patients who were unfamiliar with computer use. All patients gave informed consent to take part in the study and the study was carried out in compliance with Helsinki Declaration.

### Selection of questionnaires

Initially, contents of both generic and specific questionnaires commonly used for outcome measurement in LBP were reviewed. The candidate item sets to be used as an item bank in CAT was designed to be applicable to patients with a spectrum of LBP problems and to represent the ICF components of disability [7] and the ICF core set for LBP [25]. Another requirement was the existence of a validated Turkish version of the outcome measure to be selected. After considering these requirements, 4 questionnaires were selected: The Oswestry Disability Index (ODI), the Roland Morris Low Back Pain Disability Questionnaire (RDQ), the World Health Organization Disability Assessment Schedule (WHODAS II), and the Nottingham Health Profile (NHP).

The WHODAS II was developed by the World Health Organisation to assess functioning and disability [26]. Based on the ICF model, it is a 36-item, generic, multidimensional questionnaire which is used for measuring the levels of disability in terms of activities and participation. It includes six domains: understanding and communicating (6 items), getting around (5 items), self care (4 items), getting along with others (5 items), household and work activities (8 items), and participation in society (8 items). It has a 5-point rating scale on all items in which "1" indicates no difficulty and "5" indicates extreme difficulty or inability to perform the activity. Raw scores are transformed into standardized scores. The total score and subscale scores range between 0–100, with higher scores reflecting greater disability. A previously adapted Turkish version of the WHODAS II instrument was used [27].

The Oswestry Disability Index (ODI) is a self-completed questionnaire designed for assessing the degree of functional limitation and pain in patients with LBP [28]. It includes 10 items (pain intensity, personal care, lifting, walking, sitting, standing, sleeping, sex life, social life, and travelling), each of which has 6 ordinal responses. The scale has a total score ranging between 0 and 100 with a high score showing higher disability. The Turkish adaptation was used in this study [29].

The Roland & Morris Disability Questionnaire (RDQ) is a self-completed questionnaire designed to assess physical disability due to LBP [28]. It includes 24 items, each with a dichotomous response category of yes or no. The scale has a total score ranging between 0 and 24 with a high score showing higher disability. The Turkish version of the RMDQ was used in this study [30].

The Nottingham Health Profile (NHP) is a generic health status measure developed to record the perceived distress of patients in physical, emotional and social domains [31]. It comprises 38 statements (answered 'yes' or 'no') in six sections: physical mobility (8 items), pain (8 items), sleep (5 items), emotional reactions (9 items), social isolation (5 items) and energy level (3 items). The Turkish version of NHP was used [32]. In this version the score on each section of the NHP is the percentage of items affirmed by the respondent (that is, the number of 'yes' responses multiplied by 100 and divided by the number of items in that section). Possible scores could range from 0 to 100, with a higher score indicating greater distress.

As seen above, response options and corresponding scores of items across the scales were different. While the items of WHODAS II and ODI were polytomous, those of RDQ and NHP were dichotomous.

The contents of these questionnaires were examined by the investigators regarding their links to the categories of ICF components [6,33] and also the ICF LBP core set [25]. This examination revealed that some of the items had links with categories covered in both "body functions" and "activities and participation." Another issue at this stage was that some ICF core set categories from the body functions component (mobility and stability of joint functions, muscle power and muscle tone), and one category from the activities and participation component (toileting), were not covered in the contents of the questionnaires. However, as uncovered body function categories require a physical examination, it was impossible to include them in a self-report questionnaire. Regarding the toileting activity, which was the only "activities and participation" category missing, the investigators decided that it was not an essential deficit as most of the components of toileting activity such as sitting, rising from sitting position and dressing were already covered in other items. Furthermore none of the other questionnaires used in LBP were assessing toileting activity. Thus the four chosen scales gave 108 items as candidate items for the item bank.

### Data analysis

#### Initial unidimensionality testing

The 108 items were submitted to an exploratory factor analysis (EFA) for categorical data using weighted least square methods [34] to investigate the dimensionality of the item set. Model fit was evaluated using the root-mean-square error of approximation (RMSEA) that accounts for model parsimony. RMSEA values < 0.08 suggest adequate fit; values < 0.05 indicate good fit [10].

When more than one dimension was found according to the results of EFA, separate item sets were constructed and named. Items, whose factor loadings below 0.40, were eliminated from the item set(s) [11]. After the determination of the dimensions of the total item set by EFA, the next step was to calibrate these items onto their appropriate dimensions using an IRT model.

#### IRT model selection

The Rasch model, sometimes referred to as the one-parameter IRT model, produces latent trait person estimates that are independent of the distribution of the population, and item difficulty estimates which are independent of the ability of the person [35]. These are requirements for obtaining interval scale estimates [36]. This then allows, for example, the calculation of person change scores from what was originally ordinal data [37]. Master's partial credit model (PCM) is an extension of the Rasch dichotomous model which can accommodate items with different response categories, such as those proposed for the LBP item bank [38]. The PCM equation, in the logit form is:

$$\ln\left(\frac{P_{nik}}{1-P_{nik-1}}\right)=\theta_{n}-b_{ik}$$

where *P*_*nik *_is the probability of person *n *affirming category *k *in item *i*, compared with an adjacent category (*k-1*); *θ*_*n *_is person ability, *b*_*ik *_is the difficulty of the *k*^*th *^threshold which is the probabilistic midpoint (i.e., 50/50) between any 2 adjacent categories in item *i*.

The resulting Rasch analysis, as with all versions of the Rasch model, is mostly concerned with testing the underlying assumptions of the model; that of the probabilistic relationship between items, unidimensionality and local independence [39]. In addition, item bias or differential item functioning can be examined.

#### Unidimensionality and local independence

The PCM is a unidimensional measurement model, therefore the assumption is that the items summed together form a unidimensional scale. There are various ways to test this assumption, and these can be thought of as a series of indicators to support the assumption. Rasch programs usually provide a principal component analysis of the residuals. The absence of any meaningful pattern in the residuals will also be deemed to support the assumption of unidimensionality. A test for unidimensionality, proposed by Smith EV [19], takes the patterning of items in the residuals, examining the correlation between items and the first residual factor, and uses these patterns to define two subsets of items (i.e., the positively and negatively correlated items). These two sets of items are then used to make separate person estimates, and, using an independent *t*-test for the difference in these estimates for each person, the percentage of such tests outside the range -1.96 to 1.96 should not exceed 5%. A confidence interval for a binomial test of proportions is calculated for the proportion of observed number of significant tests, and the lower bound should overlap the 5% expected value for the scale to be unidimensional. Given that the differences in estimates derived from the two subsets of items are normally distributed, this approach is robust enough to detect multidimensionality [40] and appears to give a test of strict unidimensionality, as opposed to essential unidimensionality [41]. In the latter case a dominant factor occurs, and although other factors exist, they are not deemed to compromise measurement.

The assumption of local independence implies that when the 'Rasch factor' has been extracted, that is, the main scale, there should be no leftover patterns in the residuals. This assumption was tested by performing a PCA analysis of the residuals obtained from PCM. If a pair of items had a residual correlation of 0.30 or more, one of the items that showed a higher accumulated residual correlation with the remaining items was eliminated [42].

#### Correct ordering of response categories

Before evaluation of item fit, where polytomous items are involved, the response categories should be examined for correct ordering. This involves the examination of the threshold pattern, the threshold being the transition point between adjacent categories. This ordering of thresholds is graphically demonstrated in the category probability curves by using the RUMM2020 software [43]. For an item with an appropriate ordering of thresholds each response option would demonstrate the highest probability of endorsement at a specific range of the scale, with successive thresholds found at increasing levels of the construct being measured. One of the most common sources of item misfit concerns respondents' inconsistent use of these response options. This results in what is known as disordered thresholds and usually, although not always, collapsing of categories where disordered thresholds occur improves overall fit to the model [44].

#### Item fit

In the current analysis, individual item fit statistic and individual person fit statistic are presented, both as residuals and as a chi square statistics. The individual item fit statistic is based on the standardised residuals (differences between the observed and expected responses divided by square root of variance and calculated for each patient for a given item). To obtain an overall statistic for an item, the standardised residuals are squared and summed over the patients. The individual item fit statistic is calculated by transforming this overall statistic to make it more nearly approximate a standard normal deviate under the hypothesis that the data fit the model. Thus, it is concluded that the deviations between the responses and the model are no more than random errors. Residuals between ± 2.5 are deemed to indicate adequate fit to the model. A person fit statistic is constructed for each person in a way similar to that of each item. A chi-square test is also available for each item. The chi-square statistics compares the difference in observed values with expected values across groups representing different ability levels (called class intervals) across the trait to be measured. Consequently, for a given item, several chi-squares are computed (the number of groups depend on sample size), and then these chi-square values are summed to give the overall chi-square for the item, with degrees of freedom being the number of groups minus 1. If the p value calculated from the overall chi-square is less than 0.05 (or Bonferroni-adjusted value) then the item is deemed to misfit to the model [45].

In addition to these individual fit statistics explained above, overall item fit statistics, overall person fit statistics and item-trait interaction statistics are presented. If the data accord to the model expectation, the mean of the overall item and the overall person fit statistics should be close to 0 and their standard deviation close to 1. A third summary fit statistics is an item-trait interaction statistics reported as a Chi-Square, reflecting the property of invariance across the trait. This statistic sums the chi-squares for individual items across all items. A significant chi-square indicates that the hierarchical ordering of the items varies across the trait, compromising the required property of invariance. A wide variety of texts are available to help the reader understand fit and the other relevant topics discussed in this article [35,45-48].

#### Differential item functioning

DIF, or item bias, can also affect fit to the model. This occurs when different groups within the sample (e.g., younger and older persons) respond in a different manner to an individual item, despite having equal levels of the underlying characteristic being measured. Therefore, this does not preclude a different score between younger and older persons, but rather indicates that, given the same level of, for example, pain, the expected score on any item should be the same, irrespective of age. Two types of DIF may be identified. One is where the group shows a consistent systematic difference in their responses to an item, across the whole range of the attribute being measured, which is referred to as uniform DIF [20]. When there is non-uniformity in the differences between the groups (e.g., differences vary across levels of the attribute), then this is referred to as non-uniform DIF. The analysis of DIF has been widely used to examine cross-cultural validity, and readers can find an explanation of the approach, including the analysis of variance-based statistical analysis used in RUMM2020 software [43], in several recent reports [21,49,50]. In the current analysis, DIF was tested by age and gender.

Thus items to be entered into the item bank are required to satisfy Rasch model expectations, be free of DIF, and meet strict unidimensionality and local independence assumptions. This applies to the 'item bank' in total.

#### Reliability

An estimate of the internal consistency reliability of the item bank was tested by Person Separation Index (PSI). This is equivalent to Cronbach's alpha [51] but has the linear transformation from the Rasch model substituted for the ordinal raw score [52].

#### Computerized adaptive testing (CAT)

Given the calibrated item bank, the next stage is to apply the CAT application. We have developed new CAT software, *Smart*CAT™ (v1.0) [53], following the logic of Thissen and Mislevy [22] during this study.

**Figure 1:**
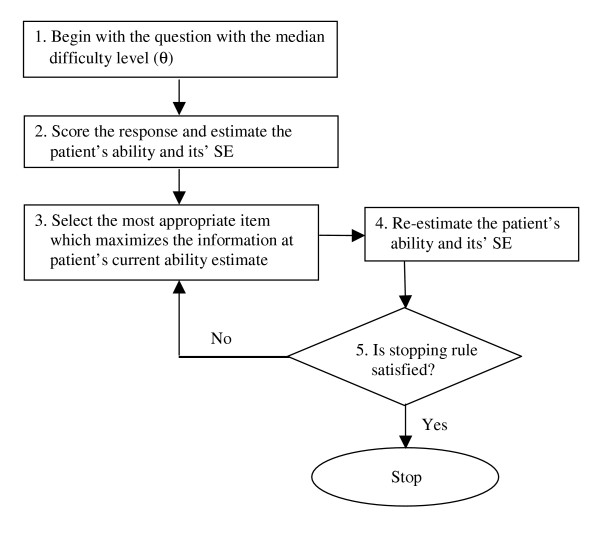
**The flow chart of the CAT algorithm used in this study**.

In CAT, when a test is administered to a patient by using a package program via the computer, the program estimates the patient's ability after each question, and then that ability estimate can be used in the selection of subsequent items. For each item, there is an item information function (centred on item difficulty in the dichotomous case), and the next item chosen is usually that which maximises this information. The items are calibrated by their difficulty levels from the item bank. Figure 1, which is adapted from Wainer et al. [54], shows the sequence of steps inherent in CAT administrations in our study. Initially, the question with the median difficulty level in the item bank is administered (Step 1) and the patient's ability level (θ_CAT_) and its standard error (SE) is estimated (Step2). The maximum likelihood estimation method with the Newton-Raphson iteration technique is used for this estimate in the current study [55,56]. Given this estimate, the next most appropriate item (which maximizes the information for the current θ estimate) is chosen (Step 3) and then presented to the patient and θ_CAT _and its SE are re-estimated (Step 4). If the predefined stopping rule (the SE of 0.5 or less) is not satisfied, Step 5 involves repeating Steps 3–4 until the stopping rule is met. When the stopping rule is satisfied, another dimension is measured or the assessment is completed.

#### Simulated and real-CAT applications

CAT was applied in two ways: A simulated and a real CAT application. In the simulated CAT, responses for 10000 patients derived from the RUMMss simulation program [57] were taken to represent the responses the patient would have given, had the item been administered in the context of a CAT. These data were simulated to meet Rasch model expectations using the item difficulty estimates from the item bank. Patient's disability level was normally distributed with a mean of 0 and standard deviation of 2. It was assumed that the mode of administration (i.e. paper and pencil which gave estimates for the item bank or the CAT application) would not substantially have affected item responses when the CAT estimated the disability level (θ_S-CAT_) and its SE for each patient. These estimations (θ_S-CAT_) were compared with the disability levels (θ_S-PCM_) generated by the simulation program using all the items based upon the original calibration using the PCM.

In the real-CAT application, 133 patients were asked to complete both a paper-and-pencil test of the full item bank, and the CAT version. Estimations from the real-CAT application (θ_R-CAT_) were compared with the disability levels generated using the response to all items analyzed with a PCM (θ_R-PCM_), with item difficulties anchored to the original calibration of 266 cases.

At the final stage, the estimates derived from the real CAT application (θ_R-CAT_) were compared with those derived from all the original questionnaires, including subscale scores, in order to demonstrate a limited form of convergent validity [14].

To summarize the approach used in this study; questionnaires that had been adapted in the Turkish language were chosen to include the ICF components of disability and the ICF categories listed in the ICF core set for LBP. The dimensionality of the total item set was explored using EFA for categorical data and the psychometric properties of the resulting item set were then evaluated by the Rasch (PCM) model [38]. The calibrations of the items which satisfied the model expectations then formed the item bank which was subsequently included in the CAT process. The CAT process involved both simulated and real (i.e. patient completed) responses. A comparison was made between the simulated CAT (θ_S-CAT_) and the original estimate provided by the simulation programme (θ_S-PCM_). A further comparison was made between the disability levels estimated from the item bank (θ_R-PCM_) and those generated using real (observed) CAT (θ_R-CAT_). And for the last stage, a form of convergent validity between the real CAT derived estimates and the scores from the original questionnaires were also examined. The response burden of the CAT process in terms of the number of items was compared to the 'paper and pencil' approach.

#### Sample size and statistical software

For the Rasch analysis it is reported that a sample size of 266 patients will estimate item difficulty, with α of 0.05, to within ± 0.3 logits [58]. This sample size is also sufficient to test for DIF where, at α of 0.05 a difference of 0.3 within the residuals can be detected for any 2 groups with β of 0.20. Bonferroni corrections are applied to both fit and DIF statistics due to the number of tests undertaken [59]. A value of 0.05 is used throughout, and corrected for the number of tests. Convergent validity between the real CAT derived estimates and the scores from the original questionnaires, including the subscales, were tested by the Spearman's correlation coefficient (r). The Intraclass correlation coefficient [ICC (2,1)] [60] and the Bland-Altman method [61] were used for evaluating the agreement between PCM and CAT derived θ estimations.

Statistical analysis was undertaken with SPSS 11.5; exploratory factor analysis with the MPlus program [34]; Rasch analysis with the RUMM2020 package [43] and the simulation were undertaken with RUMMss [57]. The CAT application used *Smart*CAT™ (v1.0) [53].

## Results

A total of 266 patients with low back pain answered 108 items from the four original questionnaires. The mean age of the patients was 52.2 years (standard deviation (SD) 12.5), 16% were men, and patients had a mean complaint time of 8.24 years (minimum: 1 month; maximum: 40 years). Prior to detailed analysis, it was observed that few patients worked (13%) and only half of the group had an active sexual life (50%). Thus a total of 6 work and sexual life related items (5 from the WHODAS II and 1 from the ODI) were removed from the item set.

### Initial unidimensionality

An Exploratory Factor Analysis (EFA) was conducted with the remaining 102 items. Due to highly negative correlations (< -0.99) with other items, three items were removed from the analysis and a new EFA was conducted with 99 items. This analysis produced a two-factor solution. When the items were examined regarding their links with the ICF categories, it was seen that items in the first dimension were related to pain, sleep, cognitive and emotional aspects of health, therefore this dimension was named as "body functions". The second dimension included items concerned with activities and participation (e.g., mobility, self-care activities, domestic life, social life), and was therefore named as "activity-participation". The factor loadings varied from 0.425 to 0.883 for the body functions and 0.413 to 0.935 for activity-participation. At this stage, none of the items loaded on both dimensions with a factor loading of 0.40 or above, but five items failed to load on either dimension, and so were removed from the item set. The RMSEA value for the two-factor solution was 0.087. Although this RMSEA value is a little high, it was concluded that the 40-item "body functions" set and the 54-item "activity-participation" set represented good starting points to create a unidimensional item bank for each construct.

### Rasch analysis

#### "Body functions" dimension

**Figure 2:**
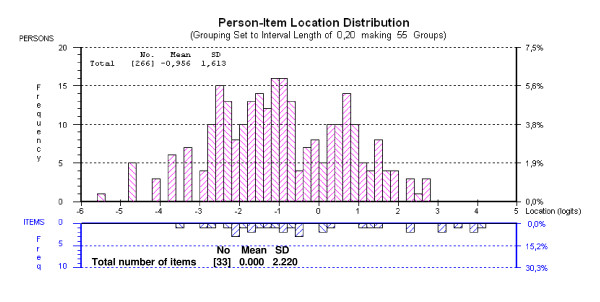
**Targeting of "Body Functions" item bank to patient disability (after collapsing of the categories)**. (n = 266).

Starting with 40 items, many of those that were polytomous displayed disordered thresholds, necessitating collapsing of categories. Following this, all items apart from "ODI 1, ODI 7, WHODAS II – 1.2, WHODAS II – 1.4, NHP 3, NHP 5 and NHP 33" were found to fit the model (given a Bonferroni adjustment fit level of 0.001) (Table 1). Overall mean item fit residual was 0.552 (SD 0.992) and mean person fit residual was -0.379 (SD 1.077). Item-trait interaction was non-significant, supporting the invariance of items (chi-square 132.64 (df = 99), p = 0.0136). The PSI was good (0.91) indicating the ability of the scale to differentiate more than 4 groups of patients [52]. Overall, the resulting 33-item item bank was not particularly well targeted. With a mean person score of -0.956, patients in this study displayed a lower average level of body functioning than the average level of the item bank (Figure 2). DIF was tested for age and gender, but all the items were free of DIF.

**Table 1:** Fit of "Body Functions" item bank to Rasch model (after rescoring) (n = 266)

**Item**	**Location**	**SE**	**Individual Item Fit Residual**	**Chi-Square Test Statistics**	**p**
**WHODAS II – 1.1. **In the last 30 days, how much difficulty did you have in concentrating on doing something for ten minutes?	1.518	0.140	0.879	8.326	0.040
**WHODAS II – 1.3. **In the last 30 days, how much difficulty did you have in analyzing and finding solutions to problems in day to day life?	3.084	0.151	0.042	0.498	0.919
**WHODAS II – 1.5. **In the last 30 days, how much difficulty did you have in generally understanding what people say?	2.245	0.153	-0.067	1.549	0.671
**WHODAS II – 1.6. **In the last 30 days, how much difficulty did you have in starting and maintaining a conversation?	3.521	0.166	-0.147	0.985	0.805
**WHODAS II – 4.1. **In the last 30 days, how much difficulty did you have in dealing with people you do not know?	3.878	0.196	0.383	3.269	0.352
**WHODAS II – 4.2. **In the last 30 days, how much difficulty did you have in maintaining a friendship?	4.110	0.221	-0.495	7.708	0.052
**WHODAS II – 4.3. **In the last 30 days, how much difficulty did you have in getting along with people who are close to you?	3.084	0.185	-1.006	1.652	0.648
**WHODAS II – 4.4. **In the last 30 days, how much difficulty did you have in making new friends?	3.953	0.208	0.021	2.977	0.395
**WHODAS II – 6.3. **In the last 30 days, how much of a problem did you have living with dignity because of the attitudes and actions of others?	2.264	0.162	0.725	1.592	0.661
**WHODAS II – 6.5. **In the last 30 days, how much have you been emotionally affected by your health condition?	0.317	0.159	-0.237	2.529	0.470
**RDQ 13. **My back is painful almost all of the time	-2.956	0.179	-0.266	3.331	0.343
**RDQ 18. **I sleep less well because of my back	-1.661	0.152	0.655	1.915	0.590
**RDQ 22. **Because of back pain, I am more irritable and bad tempered with people than usual	-2.762	0.173	-1.237	1.950	0.583
**NHP 1. **I'm tired all the time	-3.530	0.203	-0.454	1.845	0.605
**NHP 2. **I have pain at night	-2.146	0.159	0.671	3.030	0.387
**NHP 4. **I have unbearable pain	0.093	0.159	1.423	3.156	0.368
**NHP 6. **I've forgotten what it's like to enjoy myself	-0.859	0.150	-0.427	3.987	0.263
**NHP 7. **I'm feeling on edge	-1.948	0.156	-2.143	8.312	0.040
**NHP 9. **I feel lonely	-0.680	0.151	-1.264	6.513	0.089
**NHP 13. **I'm waking up in the early hours of the morning	-2.018	0.157	1.478	11.751	0.008
**NHP 15. **I'm finding it hard to make contact with people	1.062	0.180	-1.854	8.311	0.040
**NHP 16. **The days seem to drag	-0.427	0.153	-2.530	9.019	0.029
**NHP 20. **I lose my temper easily these days	-2.018	0.157	-0.733	0.632	0.889
**NHP 21. **I feel there is nobody that I am close to	-0.512	0.152	-1.411	8.458	0.037
**NHP 22. **I lie awake for most of the night	-1.129	0.150	-1.092	5.465	0.141
**NHP 23. **I feel as if I'm losing control	-0.904	0.150	-1.229	0.889	0.828
**NHP 28. **I'm in constant pain	-2.208	0.160	-0.693	3.152	0.369
**NHP 29. **It takes me a long time to get to sleep	-1.590	0.152	-0.154	6.140	0.105
**NHP 30. **I feel I am a burden to people	-0.420	0.153	-0.630	0.055	0.997
**NHP 31. **Worry is keeping me awake at night	-1.200	0.150	-2.118	5.686	0.128
**NHP 32. **I feel that life is not worth living	0.189	0.160	-0.895	1.724	0.632
**NHP 34. **I'm finding it hard to get along with people	1.249	0.186	-0.187	3.736	0.291
**NHP 37. **I wake up feeling depressed	-1.601	0.152	-1.579	2.496	0.476

WHODAS II: World Health Organization Disability Assessment Schedule II, RDQ: Roland Morris Low Back Pain Disability Questionnaire, NHP: Nottingham Health Profile, SE: Standard Error

Finally, using the PCA of residuals obtained from PCM, taking the highest positively and negatively correlated items to the first residual factor to make two subsets, no significant difference in person estimates (t = 6.8%; CI 4.2%–9.4%) was found between the two subsets, thus supporting the unidimensionality of the item bank. When the assumption of local independence was examined, there was no pair of items which had a residual correlation of 0.30 or more.

#### "Activity-participation" dimension

Starting with 54 items, many polytomous items displayed disordered thresholds, necessitating collapsing of categories. Following this, items "ODI 2, ODI 3 and ODI 5" did not fit the model (given a Bonferroni adjustment fit level of 0.001) and were removed. Overall mean item fit residual was -0.239 (SD 1.411) and mean person fit residual was -0.412 (SD 0.959). Item-trait interaction was non-significant, suggesting the invariance of items (chi-square 204.46 (df = 153), p = 0.0035). The PSI was good (0.94) indicating the ability of the scale to differentiate more than 4 groups of patients [52].

**Figure 3:**
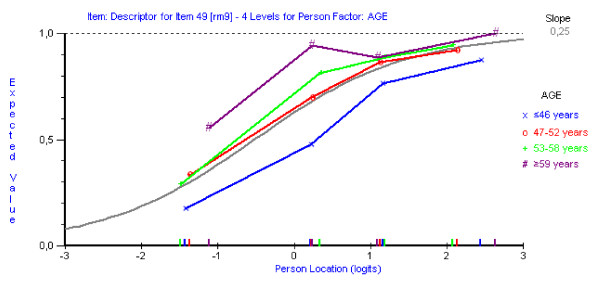
**Differential Item Functioning for item RDQ 9 "I get dressed more slowly than usual because of my back" by age**. (n = 266).

DIF was tested for age and gender. Only item "RDQ 9 – I get dressed more slowly than usual because of my back" showed a uniform DIF in terms of age, but the other items were free of DIF. As shown in Figure 3, older patients perceived dressing to be more difficult than young patients across the whole range of the attribute being measured. However, the item was thought to be important for patients and was retained in the item bank.

Finally, PCA analysis of residuals obtained from PCM, taking the highest positively and negatively correlated items to create two subsets of items showed that "WHODAS II – 5.2 and NHP 17" violated the unidimensionality assumption, and thus were removed from the item bank. Following this modification, good fit to the Rasch model was attained (Table 2) for the remaining 49-item bank with a non-significant item-trait chi-square, supporting the invariance of items across the scale (chi-square 190.712 (df = 147), p = 0.009). Overall mean item fit residual was -0.236 (SD 1.375) and person fit residual of -0.397 (SD 0.937). The PSI was 0.94, indicating the ability of the scale to differentiate at least 4 groups of patients [52].

**Table 2:** Fit of "activity-participation" item bank to Rasch model (after rescoring) (n = 266)

**Item**	**Location**	**SE**	**Individual Item Fit Residual**	**Chi-Square Test Statistics**	**p**
**WHODAS II – 2.1. **In the last 30 days, how much difficulty did you have in standing for long periods such as 30 minutes?	0.132	0.116	2.141	6.255	0.100
**WHODAS II – 2.2. **In the last 30 days, how much difficulty did you have in standing up from sitting down?	1.846	0.152	-1.500	2.695	0.441
**WHODAS II – 2.3. **In the last 30 days, how much difficulty did you have in moving around inside your home?	2.752	0.146	-0.579	3.289	0.349
**WHODAS II – 2.4. **In the last 30 days, how much difficulty did you have in getting out of your home?	1.907	0.162	-1.600	5.242	0.155
**WHODAS II – 2.5. **In the last 30 days, how much difficulty did you have in walking a long distance such as a kilometer (or equivalent)?	0.110	0.114	-0.115	1.716	0.633
**WHODAS II – 3.1. **In the last 30 days, how much difficulty did you have in washing your whole body?	4.182	0.149	-0.844	2.264	0.519
**WHODAS II – 3.2. **In the last 30 days, how much difficulty did you have in getting dressed?	4.376	0.145	-0.638	1.780	0.619
**WHODAS II – 3.3. **In the last 30 days, how much difficulty did you have in eating?	4.163	0.156	1.320	0.275	0.965
**WHODAS II – 3.4. **In the last 30 days, how much difficulty did you have in staying by yourself for a few days?	2.540	0.130	2.009	7.187	0.066
**WHODAS II – 5.3. **In the last 30 days, how much difficulty did you have in doing most important households tasks well?	1.882	0.146	-1.405	4.658	0.199
**WHODAS II – 5.4. **In the last 30 days, how much difficulty did you have in getting all the household work done that you needed to do?	-0.038	0.115	-0.758	5.290	0.152
**WHODAS II – 5.5. **In the last 30 days, how much difficulty did you have in getting your household work done as quickly as needed?	-0.144	0.120	-0.608	2.190	0.534
**WHODAS II – 6.1. **In the last 30 days, how much of a problem did you have in joining in community activities (for example, festivities, religious or other activities) in the same way as anyone else can	1.911	0.119	0.471	0.456	0.928
**WHODAS II – 6.2. **In the last 30 days, how much of a problem did you have because of barriers or hindrances in the world around you?	3.722	0.142	1.391	6.266	0.099
**WHODAS II – 6.8. **In the last 30 days, how much of a problem did you have in doing things by yourself for relaxation or pleasure?	2.115	0.141	-0.986	0.793	0.851
**ODI 4. **Walking	3.082	0.106	0.361	7.119	0.068
**ODI 6. **Standing	0.651	0.104	0.447	4.730	0.193
**ODI 9. **Social Life	1.578	0.102	1.585	11.801	0.008
**ODI 10. **Travelling	0.216	0.137	-1.365	1.964	0.580
**RDQ 1. **I stay at home most of the time because of my back	-0.396	0.156	-0.836	2.526	0.471
**RDQ 2. **I change position frequently to try to get my back comfortable	-2.904	0.274	-0.207	2.106	0.551
**RDQ 3. **I walk more slowly than usual because of my back	-1.705	0.198	-1.807	9.433	0.024
**RDQ 4. **Because of my back, I am not doing any jobs that I usually do around the house	-1.064	0.173	-0.307	2.586	0.460
**RDQ 5. **Because of my back, I use handrail to get upstairs	-1.862	0.205	-1.482	4.863	0.182
**RDQ 6. **Because of my back, I lie down to rest more often	-1.745	0.200	-0.001	1.760	0.624
**RDQ 7. **Because of my back, I have to hold on to something to get out of an easy chair	-0.482	0.158	0.522	2.695	0.441
**RDQ 8. **Because of my back, I try to get other people to do things for me	-0.252	0.153	1.712	8.550	0.036
**RDQ 9. **I get dressed more slowly than usual because of my back	-0.522	0.159	-0.079	1.594	0.661
**RDQ 10. **I only stand up for short periods of time because of my back	-2.219	0.225	-1.558	4.238	0.237
**RDQ 11. **Because of my back, I try not to bend or kneel down	-3.023	0.284	-0.823	2.227	0.527
**RDQ 12. **I find it difficult to get out of chair because of my back	-0.647	0.162	-0.527	3.033	0.387
**RDQ 14. **I find it difficult to turn over in bed because of my back	-1.157	0.177	-1.006	4.851	0.183
**RDQ 16. **I have trouble putting on my sock (or stockings) because of the pain in my back	-0.753	0.164	-0.316	2.821	0.420
**RDQ 17. **I can only walk short distances because of my back pain	-0.745	0.164	-2.068	8.215	0.042
**RDQ 19. **Because of my back pain, I get dressed with the help of someone else	3.879	0.253	-0.611	4.508	0.212
**RDQ 21. **I avoid heavy jobs around the house because of my back	-1.734	0.199	0.091	1.217	0.749
**RDQ 23. **Because of my back, I go upstairs more slowly than usual	-2.556	0.247	-1.294	3.386	0.336
**RDQ 24. **I stay in bed most of the time because of my back	1.959	0.157	0.786	7.979	0.046
**NHP 8. **I find it painful to change position	-1.594	0.193	-0.478	5.599	0.133
**NHP 11. **I find it hard to bend	-1.954	0.210	-1.284	2.197	0.533
**NHP 12. **Everything is an effort	-0.805	0.166	-1.470	7.025	0.071
**NHP 18. **I find it hard to reach for things	-2.090	0.217	-1.710	3.055	0.383
**NHP 19. **I'm in pain when I walk	-1.740	0.200	-1.263	3.802	0.284
**NHP 24. **I'm in pain when I'm standing	-2.701	0.257	-0.611	0.774	0.856
**NHP 25. **I find it hard to get dressed by myself	1.174	0.146	1.298	2.948	0.400
**NHP 26. **I soon run out of energy	-2.134	0.220	5.455	1.989	0.575
**NHP 27. **I find it hard to stand for long (e.g., at the kitchen sink, waiting in a line)	-3.334	0.314	-1.092	1.178	0.758
**NHP 36. **I'm in pain when going up or down stairs	-2.459	0.240	-1.310	5.191	0.158
**NHP 38. **I'm in pain when I'm sitting	-1.417	0.186	1.374	2.394	0.495

WHODAS II: World Health Organization Disability Assessment Schedule II, ODI: Oswestry Disability Index, RDQ: Roland Morris Low Back Pain Disability Questionnaire, NHP: Nottingham Health Profile, SE: Standard Error

The unidimensionality of the item bank was supported by the individual t-test showing 7.5% of tests as significant (CI 4.9%–10.2%). When the assumption of local independence was examined, there was no pair of items having residual correlation of 0.30 or more.

**Figure 4:**
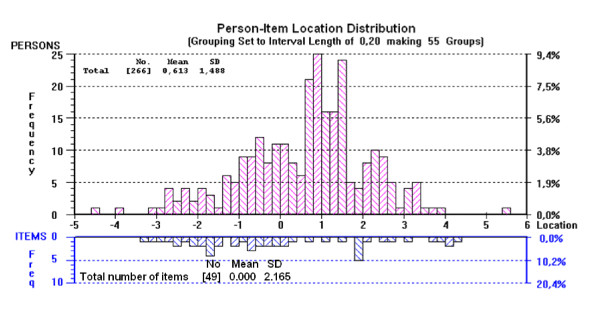
**Targeting of "Activity-Participation" item bank to patient disability (after collapsing of the categories)**. (n = 266).

Overall, the item bank was reasonably targeted in that the measurement, expressed through the distribution of the location, covered almost all disability levels of patients across the trait (Figure 4). With a mean person score of 0.613, patients in this study displayed a slightly higher level of activity limitation- participation restriction than the average of the item bank.

#### Reliability

Internal consistencies of the item banks were adequate at the dimension level with Cronbach's alphas of 0.91 and 0.93 and the PSI values of 0.91 and 0.94 for the first and second item banks, respectively.

### CAT development and simulation

#### Simulation results

For the simulated CAT application, 95% ranges of agreement between θ_S-CAT _and θ_S-PCM _according to Bland-Altman approach were -0.695 to 1.174 for the body functions and -1.038 to 1.213 for activity-participation dimensions. Furthermore, 8566 of the 9056 and 9456 of the 9916 converged estimates were also within the 95% limits of agreement for the first and second dimensions, respectively. The θ_S-PCM _and θ_S-CAT _correlated well (for the first dimension *r *= 0.96 and ICC = 0.95 and for the second dimension 0.97 and 0.96, respectively). The initial CAT setting used a median of 19 items for body function, and 15 items for activity-participation dimensions.

#### Real CAT results

A total of 133 patients with low back pain completed 108 items from the four original questionnaires and the CAT version. The mean age of these patients was 53.0 years (standard deviation (SD) 13.9), 19.5% were men, and patients had a mean complaint time of 7.0 years (minimum: 1 month; maximum: 30 years).

For the real initial CAT application, 95% ranges of agreement according to Bland-Altman approach were -0.487 to 0659 and -0.734 to 0.776 for the body functions and activity-participation dimensions, respectively. A total of 126 of the 133 patients were within the 95% limits of agreement for body functions, and 126 of the 133 patients were within the 95% limits of agreement for the activity-participation dimension. The ICC (2,1) values were 0.98 and 0.97, respectively. The CAT used median of 19 and 14 items to estimate θ for the body functions and activity-participation, respectively. θ_R-PCM _and θ_R-CAT _correlated well for the body functions and activity-participation dimensions (*r *= 0.98 and *r *= 0.97, respectively).

#### Respondent burden

As would be expected, respondent burden was substantially greater for those who completed all items in the scales, in comparison with those for whom scores were estimated using CAT. CAT assessments initially reduced the number of items administered to 19 and 14 per patient for the first and second item banks. This reduction in number of items administered translated into estimated reductions in response times from an average of 15 to 6 minutes.

#### Reducing the burden further

The initial CAT application included a standard error of 0.50 or less as a stopping rule. We increased the standard error to 0.55 and 0.60 to test if this further reduced the burden. As a result, the average number of items administered fell to 15 and 12 for the body functions dimension, and to 12 and 10 for the activity-participation dimension respectively, for these increased standard errors.

Finally, a form of convergent validity between the estimates from the real CAT (θ_R-CAT_) and those derived from all the original questionnaires was examined. Most of the NHP sections, such as sleep, pain, social isolation and emotional reactions had high correlations (> 0.60) with the θ_R-CAT _body functions estimates. Similarly, WHODAS II self-care and getting around sections, NHP physical mobility section, RDQ and ODI total scores had high correlations (> 0.70) with the θ_R-CAT _activity-participation estimates (Table 3).

**Table 3:** Convergent validity between θ_R-CAT _and subscale and total scores of the four original questionnaires.

**Total/section scores**	**θ_R-CAT _Body Functions**	**θ_R-CAT _Activity-Participation**
WHODAS II – Understanding and communicating	0.587**	0.396**
WHODAS II – Getting around	0.437**	0.801**
WHODAS II – Self care	0.391**	0.642**
WHODAS II – Getting along with people	0.382**	0.321**
WHODAS II – Getting along with people (without sexual activities item)	0.260*	0.071
WHODAS II – Life activities (without work items)	0.423**	0.647**
WHODAS II – Participation in society	0.549**	0.697**
WHODAS II – Total (without work and sexual items)	0.667 **	0.820**
ODI	0.531**	0.724**
RDQ	0.639**	0.748**
NHP Energy	0.595**	0.424*
NHP Pain	0.659**	0.547**
NHP Emotional	0.892**	0.422**
NHP Sleep	0.737**	0.424**
NHP Social Isolation	0.809**	0.422**
NHP Physical Mobility	0.510**	0.708**

*: ≤ 0.01, **: ≤ 0.001

WHODAS II: World Health Organization Disability Assessment Schedule II, ODI: Oswestry Disability Index, RDQ: Roland Morris Low Back Pain Disability Questionnaire, NHP: Nottingham Health Profile.

Expressed as Spearman's correlation (n = 91).

## Discussion

This study is the first to explore the potential for applying CAT in the assessment of ICF related disability for outcome measurement in LBP. Using a combination approach of EFA and Rasch analysis, based upon the disability definition in the ICF, together with new developments in CAT software, we have been able to show that items can be calibrated onto a single metric and that they can be used to provide the basis of a CAT application which map on to the ICF. In this way, a simple, precise estimate of the person's ability can be determined and, given the use of the Rasch model, one that is interval scaled. Furthermore, the combination of items from different questionnaires makes a wider 'ruler' of ability than any single scale, reducing the risk of floor and ceiling effects, and providing continuity of measurement across the acute-community divide.

The development and implementation of such an approach has raised, and continues to raise several developmental and application challenges. At the conceptual level for example, not all items within the ICF core set are accommodated within our item banks [25]. Consequently, further expansion to make these item banks inclusive, at least of the brief core sets, would be advantageous. However, there is no guarantee that additional items would satisfy strict unidimensional requirements as there is no empirical evidence to support the dimensionality of the published core sets. It is also true to say that the way in which tasks are operationalised in some scales can reflect both cognitive and physical components, and can potentially straddle both body functions and activities within the ICF categorisation. The task of developing a measurement system to map onto the ICF is thus an ongoing challenge, and the current study offers one potential way of providing measurement that facilitates an ICF based CAT approach. The grouping of items into body functions and activity-participation is based upon rigorous tests of unidimensionality but, for example, the latter does not attempt to separate activities from participation. Indeed there is still considerable debate about the distinction between activities and participation as defined by the ICF. A recent paper has suggested that these need further differentiation into 'acts', 'tasks' and 'societal involvement' [62].

We have adopted rigorous tests of unidimensionality as there is evidence that even small deviations from this can lead to substantive and significant differences in person estimates [40]. CAT would be particularly vulnerable to this influence as only a relatively small set of items are administered. Even then, we need to gather more data to undertake a confirmatory factor analysis on the final sets of items to have greater confidence in the unidimensionality of the item banks. An EFA approach was used because traditional factor analysis may overestimate the number of factors and underestimate the factor loadings when analyzing skewed categorical data [34]. Nevertheless, our indicator of unidimensionality (RMSEA) for the item banks was higher than we would have wanted, and suggests some fragility in the dimensionality of the structure.

In Turkey there is an educational and income gradient by age, including illiteracy and a lack of computer experience [63]. Consequently most of the patients required help with the CAT application. The computer set up was traditional, including a mouse, and touch screen technology may have improved independence for some, and is an obvious next step. The illiteracy problem is likely to remain for another 20 years or so, and so this is a particular challenge to CAT application in Turkey and other countries where there are similar problems, whereas possibly not so much in northern European countries or the USA. Nevertheless, despite these problems, internet based CAT applications, where patients can log in, should offer further opportunities for the community-based follow-up.

There are further technical issues which require further thought and development. From the simulated data it was not possible to obtain an estimate of the persons' body functions or activity-participation dimensions in all cases. The CAT application failed to converge in 9.4% and 0.8% of cases for the first and second dimensions, respectively. This is a known problem with the Newton Raphson algorithm which was used in the current study, but the next version of *Smart*CAT™ will include the modified maximum likelihood estimation procedure which should eliminate this problem [64,65]. This will leave only the estimate of extreme persons (i.e. at the floor or ceiling of the entire item bank) where additional information will be required to obtain a person estimate. Currently this was obtained from the RUMM2020 programme as the person estimate for extremes in the item bank calibration [43]. The actual number of extreme cases was low with none in the body functions and 0.01% in the activity-participation dimensions of the persons in the real CAT application. Furthermore, only 1 of 133 real CAT applications failed to converge.

The number of cases used in the current study is lower than the average by CAT standards. Previous published work on CAT has been based on sample sizes ranging from less than one hundred to several thousand cases [9,11,14,18,66]. Some of this variability may be due to the use of different IRT models as the basis of this work. Generally the Rasch model is far less demanding in terms of sample size than other IRT models [67], although it is much more demanding in terms of quality of data as it requires the scales to satisfy conjoint theory axioms [37]. The key issue is the degree of precision required of the person estimate, and this raises further interesting issues as to whether this might vary across different diagnoses and situations, for example, where estimates might be used as the basis of clinical management decisions (e.g. to start a particular treatment).

It is known that each pair of adjacent categories in the polytomous item serves as a single dichotomous item so, the polytomous item bank makes more contribution to the test information function than the dichotomous item bank. Also, the information is typically distributed across a wider range of the trait being measured when polytomous items contribute to item banks. For this reason, even when there is a relatively small item bank with polytomous items, CAT works well [68]. Since, our item banks were relatively small and most of the items in item banks were dichotomous, the number of items used to estimate the thetas with SE < 0.5 was higher in our CAT application than other CATs [8-10,12,13,69]. However, Haley et al. [70] achieved the same SE of 0.5 with 20-item CAT application and another study [66] also concluded that a 20-item adapted test was successful in achieving accurate estimates of physical functioning scores and age-based centiles. These findings were similar to the present study in terms of number of items administered and precision of the estimated theta.

## Conclusion

Using a combination approach of EFA and Rasch analysis this study has shown that it is possible to calibrate items onto a single metric in a way that can be used to provide the basis of a CAT application. Recent applications of CAT in other medical outcomes suggest that many others are working on these issues at the present time, and we could expect to see a rapid growth in the scientific basis and the ease of application during the coming years [71]. All these developments mean that at the present time, there is the opportunity to obtain a wide variety of information to evaluate the biopsychosocial model in its more complex forms, without increasing the burden of information collection for patients. Else, it will be possible to minimize the burden of data collection further compared with existing data collection protocols. Both scenarios will be based upon scientifically rigorous measurement which offers greater breadth of measurement than the traditional single scale approach.

## Competing interests

The authors declare that they have no competing interests.

## Authors' contributions

All authors participated in planning and design of the study. AHE and DÖ contributed to data extraction, analysis of the data, item banking, development of the CAT program, interpretation of the results and drafted the manuscript. AAK and ŞK contributed to selection of scales, item banking, interpretation of the results and drafted the manuscript. AT contributed to analysis of the data, interpretation of the results and drafted the manuscript. All authors read, revised and finally approved the final manuscript.

## Pre-publication history

The pre-publication history for this paper can be accessed here:



## References

[B1] Ekman M, Jonhagen S, Hunsche E, Jonsson L (2005). Burden of illness of chronic low back pain in Sweden: a cross-sectional, retrospective study in primary care setting. Spine.

[B2] Dagenais S, Caro J, Haldeman S (2008). A systematic review of low back pain cost of illness studies in the United States and internationally. Spine J.

[B3] Guzmán J, Esmail R, Karjalainen K, Malmivaara A, Irvin E, Bombardier C (2001). Multidisciplinary rehabilitation for chronic low back pain: systematic review. BMJ.

[B4] Deyo RA, Battie M, Beurskens AJ, Bombardier C, Croft P, Koes B, Malmivaara A, Roland M, Von Korff M, Waddell G (1998). Outcome measures for low back pain research. A proposal for standardized use. Spine.

[B5] Katz NJ (2003). Measures of adult back and neck function. Arthritis Rheum.

[B6] Sigl T, Cieza A, Brockow T, Chatterji S, Kostanjsek N, Stucki G (2006). Content comparison of low back pain-specific measures based on the International Classification of Functioning, Disability and Health (ICF). Clin J Pain.

[B7] World Health Organization (2001). International Classification of Functioning, Disability and Health: ICF Geneva.

[B8] Jette AM, Haley SM, Tao W, Ni P, Moed R, Meyers D, Zurek M (2007). Prospective evaluation of the AM-PAC-CAT in outpatient rehabilitation settings. Phys Ther.

[B9] Kopec JA, Badii M, McKenna M, Lima VD, Sayre EC, Dvorak M (2008). Computerized adaptive testing in back pain: validation of the CAT-5D-QOL. Spine.

[B10] Hart DL, Mioduski JE, Stratford PW (2005). Simulated computerized adaptive tests for measuring functional status were efficient with good discriminant validity in patients with hip, knee, or foot/ankle impairments. J Clin Epidemiol.

[B11] Fliege H, Becker J, Walter OB, Bjorner JB, Klapp BF (2005). Development of computer-adaptive test for depression (D-CAT). Qual Life Res.

[B12] Hart DL, Mioduski JE, Werneke MW, Stratford PW (2006). Simulated computerized adaptive test for patients with lumbar spine impairments was efficient and produced valid measures of function. J Clin Epidemiol.

[B13] Hart DL, Cook KF, Mioduski JE, Teal CR, Crane PK (2006). Simulated computerized adaptive test for patients with shoulder impairments was efficient and produced valid measures of function. J Clin Epidemiol.

[B14] Kocalevent RD, Rose M, Becker J, Walter OB, Fliege H, Bjorner JB, Kleiber D, Klapp BF An evaluation of patient-reported outcomes found computerized adaptive testing was efficient in assessing stress perception. J Clin Epidemiol.

[B15] Cook KF, Teal CR, Bjorner JB, Cella D, Chang CH, Crane PK, Gibbons LE, Hays RD, McHorney CA, Ocepek-Welikson K, Raczek AE, Teresi JA, Reeve BB (2007). IRT health outcomes data analysis project: an overview and summary. Qual Life Res.

[B16] Rose M, Bjorner JB, Becker J, Fries JF, Ware JE Evaluation of a preliminary physical function item bank supported the expected advantages of the Patient-Reported Outcomes Measurement Information System (PROMIS). J Clin Epidemiol.

[B17] Lord FM, Holtzman WH (1970). Some test theory for tailored testing. Computer-assisted instruction, testing and guidance.

[B18] Ware JE, Kosinski M, Bjorner JB, Bayliss MS, Batenhorst A, Dahlöf CGH, Teper S, Dowson A (2003). Applications of computerized adaptive testing (CAT) to the assessment of headache impact. Qual Life Res.

[B19] Smith EV (2002). Detecting and evaluation the impact of multidimensionality using item fit statistics and principal component analysis of residuals. J Appl Meas.

[B20] Teresi JA, Kleinman M, Ocepek-Welikson K (2000). Modern psychometric methods for detection of differential item functioning: application to cognitive assessment measures. Stat Med.

[B21] Hagquist C, Andrich D (2004). Is the sense of coherence-instrument applicable on adolescents? A latent trait analysis using Rasch modelling. Pers Indiv Differ.

[B22] Thissen D, Mislevy RJ (2000). Testing algorithms. Computer adaptive testing.

[B23] Bjorner JB, Chang C, Thissen D, Reeve BB (2007). Developing tailored instruments: item banking and computerized adaptive assessment. Qual Life Res.

[B24] Rasch G (1960). Probabilistic models for some intelligence and attainment tests.

[B25] Cieza A, Stucki G, Weigl M, Disler P, Jackel W, Linden S Van der, Kostanjsek N, De Bie R (2004). ICF core sets for low back pain. J Rehabil Med.

[B26] World Health Organisation Disability Assessment Schedule II. http://www.who.int/icidh/whodas/.

[B27] Ulug B, Ertugrul A, Gogus A, Kabakcý E (2001). Yetiyitimi degerlendirme cizelgesinin (WHODAS II) sizofreni hastalarýnda gecerlilik ve güvenilirligi. Turk Psikiyatr Derg.

[B28] Roland M, Fairbank J (2000). The Roland-Morris Questionnaire and the Oswestry Disability Questionnaire. Spine.

[B29] Yakut E, Düger T, Oksüz C, Yörükan S, Ureten K, Turan D, Fýrat T, Kiraz S, Krd N, Kayhan H, Yakut Y, Güler C (2004). Validation of the Turkish version of the Oswestry Disability Index for patients with low back pain. Spine.

[B30] Küçükdeveci AA, Tennant A, Elhan AH, Niyazoglu H (2001). Validation of the Turkish Version of the Roland-Morris Disability Questionnaire for Use in Low Back Pain. Spine.

[B31] European Group for Quality of Life Assessment and Health Measurement (1993). European Guide for Nottingham Health Profile Surrey.

[B32] Küçükdeveci A, McKenna SP, Kutlay S, Gürsel Y, Whalley D, Arasýl T (2000). The development and psychometric assessment of the Turkish version of the Nottingham Health Profile. Int J Rehabil Res.

[B33] Cieza A, Stucki G (2005). Content comparison of health-related quality of life (HRQOL) instruments based on the international classification of functioning, disability and health (ICF). Qual Life Res.

[B34] MPlus User's Guide.

[B35] Andrich D (1988). Rasch Models for Measurement.

[B36] Karabatsos G (2001). The Rasch model, additive conjoint measurement, and new models of probabilistic measurement theory. J Appl Meas.

[B37] Perline R, Wright BD, Wainer H (1979). The Rasch model as additive conjoint measurement. Appl Psychol Measure.

[B38] Masters G (1982). A Rasch model for partial credit scoring. Psychometrika.

[B39] Gustafsson JE (1980). Testing and obtaining fit of data to the Rasch model. Brit J Math Stat Psychol.

[B40] Tennant A, Pallant JF (2006). Unidimensionality Matters. Rasch Measure Trans.

[B41] Stout WF (1990). A new item response theory modelling approach with applications to unidimensionality assessment and ability estimation. Psychometrika.

[B42] Wright BD (1996). Local dependency, correlations and principal components. Rasch Meas Trans.

[B43] Andrich D, Lyne A, Sheridan B, Luo G (2003). RUMM2020. Rasch Unidimensional Measurement Models Software.

[B44] Pallant JF, Tennant A (2007). An introduction to the Rasch measurement model: an example using the hospital anxiety and depression scale (HADS). Br J Clin Psychol.

[B45] Tennant A, Conaghan PG (2007). The Rasch measurement model in rheumatology: what is it and why use it? When should it be applied, and what should one look for in a Rasch paper?. Arthritis Rheum.

[B46] Molenaar IW, Fischer GH, Molenaar IW (1995). Estimation of item parameters. Rasch models: foundations, recent developments, and applications.

[B47] Smith EV, Smith RM (2004). Introduction to Rasch measurement.

[B48] Wilson M (2005). Constructing measures.

[B49] Tennant A, Penta M, Tesio L, Grimby G, Thonnard JL, Slade A, Lawton G, Simone A, Carter J, Lundgren-Nilsson A, Tripolski M, Ring H, Biering-Sorensen F, Marincek C, Burger H, Phillips S (2004). Assessing and adjusting for cross-cultural validity of impairment and activity limitation scales through differential item functioning within the framework of the Rasch model: the PRO-ESOR project. Med Care.

[B50] Lawton G, Lundgren-Nilsson A, Biering-Sorensen F, Tesio L, Slade A, Penta M, Grimby G, Ring H, Tennant A (2006). Cross-cultural validity of FIM in spinal cord injury. Spinal Cord.

[B51] Cronbach LJ (1951). Coefficient alpha and the internal structure of tests. Psychometrika.

[B52] Fisher WP (1992). Reliability statistics. Rasch Measure Trans.

[B53] Öztuna D, Öztuna E, Elhan AH (2008). *Smart*CAT. (Personal communication – web site under construction).

[B54] Wainer H, Dorans NJ, Eignor D, Flaugher R, Green BF, Mislevy RJ, Steinberg L, Thissen D (2000). Computerized adaptive testing A Primer.

[B55] Mathews JH, Fink KK, Mathews JH, Fink KK (2004). Solution of nonlinear equations f(x) = 0. Numerical Methods Using Matlab.

[B56] Linacre JM (1998). Estimating measures with known polytomous item difficulties. Rasch Meas Trans.

[B57] Marais I, Andrich D (2007). RUMMss. Rasch Unidimensional Measurement Models Simulation Studies Software.

[B58] Linacre JM (1994). Sample size and item calibration stability. Rasch Measure Trans.

[B59] Bland JM, Altman DG (1995). Multiple significance tests: the Bonferroni method. BMJ.

[B60] Shrout PE, Fleiss JL (1979). Intraclass Correlation: Uses in assessing rater reliability. Psycho Bull.

[B61] Bland JM, Altman DG (1986). Statistical methods for assessing agreement between two methods of clinical measurement. Lancet.

[B62] Badley EM (2008). Enhancing the conceptual clarity of the activity and participation components of the International Classification of Functioning, Disability, and Health. Soc Sci Med.

[B63] UNICEF Turkey Statistics. http://www.unicef.org/infobycountry/Turkey_statistics.html.

[B64] Vaughan DC (1992). On the Tiku-Suresh Method of Estimation. Commun Stat Theor Method.

[B65] Tiku ML (1967). Estimating Mean and Standard Deviation from Censored normal sample. Biometrika.

[B66] Haley SM, Ni P, Fragala-Pinkham MA, Skrinar AM, Corzo D (2005). A computer adaptive approach for assessing physical functioning in children and adolescents. Dev Med Child Neurol.

[B67] McHorney CA, Monahan PO (2004). Applications of Rasch Analysis in Health Care. Med Care.

[B68] Dodd BG, De Ayala RJ, Koch WR (1995). Computerized adaptive testing with polytomous items. Appl Psych Meas.

[B69] Deutscher D, Hart DL, Dickstein R, Horn SD, Gutvirtz M (2008). Implementing and integrated electronic outcomes and electronic health record process to create a foundation for clinical practice improvement. Phys Ther.

[B70] Haley SM, Ni P, Ludlow LH, Fragala-Pinkham MA (2006). Measurement precision and efficiency of multidimensional computer adaptive testing of physical functioning using the Pediatric Evaluation of Disability Inventory. Arch Phys Med Rehabil.

[B71] Ware JE, Gandek B, Sinclair SJ, Bjorner JB (2005). Item Response Theory and Computerized Adaptive Testing: Implications for Outcomes Measurement in Rehabilitation. Rehabil Psychol.

